# Early clinical course of biopsy-proven IgA vasculitis nephritis

**DOI:** 10.1186/s12887-022-03611-9

**Published:** 2022-10-04

**Authors:** Sarina Butzer, Imke Hennies, Charlotte Gimpel, Jutta Gellermann, Gesa Schalk, Sabine König, Anja K. Büscher, Anja Lemke, Martin Pohl

**Affiliations:** 1 Department of General Pediatrics, Adolescent Medicine and Neonatology, Medical Center, Faculty of Medicine, University of Freiburg, Freiburg, Germany; 2 University of Cologne, Faculty of Medicine and University Hospital Cologne, Department of Pediatrics, Cologne, Germany; 3 Department of Pediatric Kidney, Liver and Metabolic Diseases, Children’s Hospital, Hannover Medical School, Hannover, Germany; 4 Department of Pediatric Nephrology, Charité University Hospital, Berlin, Germany; 5 University Children’s Hospital Münster, Münster, Germany; 6 Department of Pediatrics II, Children’s Hospital, University of Duisburg-Essen, Essen, Germany; 7 Department of Pediatrics, University Medical Center Hamburg-Eppendorf, Hamburg, Germany

**Keywords:** IgA vasculitis, IgA nephritis, Glomerulonephritis, Nephrotic syndrome, Immunosuppressive therapy, Children

## Abstract

**Background:**

IgA vasculitis (IgAV) is the most common form of systemic vasculitis in childhood and frequently involves the kidney. A minority of patients with IgA vasculitis nephritis (IgAVN), especially those presenting with heavy proteinuria and/or kidney failure at onset, are at risk of chronic end-stage kidney disease. For deciding upon treatment intensity, knowledge of the short-term clinical course of IgAVN is needed to improve treatment algorithms.

**Methods:**

For this retrospective multicenter study, the medical records of 66 children with biopsy-proven IgAVN were reviewed. Age, gender, medical history and therapeutic interventions were recorded. Laboratory data included serum creatinine, albumin, urinary protein excretion (UPE) and glomerular filtration rate (eGFR). Threshold values were determined for each parameter, full remission was defined as no proteinuria and eGFR > 90 ml/min/1.73m^2^.

**Results:**

Median age at onset of IgAVN was 8.9 years. 14.1% of the children presented with nephrotic syndrome, 50% had an eGFR below 90 ml/min/1.73 m^2^ and 51.5% showed cellular crescents in renal histology. The treatment regimens varied notably. Forty-four patients were treated with immunosuppression; 17 patients with crescents or nephrotic syndrome were treated with corticosteroid (CS) pulse therapy. After 6 months, UPE had decreased from 3.7 to 0.3 g/g creatinine and the proportion of patients with a decreased eGFR had fallen from 50.0% to 35.5%. Thirteen children (26.5%) achieved full remission within 6 months.

**Conclusions:**

In most patients with IgAVN proteinuria decreases slowly and kidney function improves, but full remission is reached only in a minority after 6 months. Persistent heavy proteinuria in the first two months rarely developed into long-term proteinuria. Therefore, decisions for more intense treatment should take into account the course of UPE over time.

For a comparison of treatment effects, patient numbers were too small. Prospective, randomized controlled trials are necessary to clarify risk factors and the effect of immunosuppressive therapies.

**Supplementary Information:**

The online version contains supplementary material available at 10.1186/s12887-022-03611-9.

## Background

IgA vasculitis (IgAV; formerly known as Henoch-Schönlein purpura) is the most common vasculitis in childhood with a peak incidence at 4–7 years [1]. Renal involvement occurs in 30–60% of the patients [2]. The majority of these patients presents with mild symptoms and recovers without residual damage. A small percentage develops nephrotic and/or nephritic syndrome sometimes leading to permanent organ damage and the need for kidney replacement therapy [3–7]. Known risk factors for permanent renal damage are aggressive lesions in the initial kidney biopsy, the presence of nephrotic and/or nephritic syndrome, hypertension and a relapsing course of the disease [3, 4, 6, 8, 9]. However, it is still not clear how intensive treatment should be. A 5-year kidney survival of 83% and an even lower 10-year kidney overall survival of 73% [4], as well as a high risk of dialysis in selected patients [5] might justify immunosuppressive therapy. On the other hand, patients with severe initial kidney involvement can achieve complete remission without any intervention [10]. Due to the lack of evidence-based treatment strategies and the unpredictable individual prognosis, various therapeutic regimens exist, ranging from immunosuppressive monotherapy to diverse combinations of prednisolone, mycophenolate mofetil (MMF), cyclosporine A (CSA), cyclophosphamide (CYC), azathioprine (AZA), rituximab and plasmapheresis. Early treatment initiation appears to be decisive for therapeutic success [11–15]. In order to describe the short-term renal course, we evaluated 66 patients with biopsy-proven IgA vasculitis nephritis (IgAVN; formerly known as Henoch-Schönlein purpura nephritis) and highly heterogeneous treatment strategies. Clinical symptoms, proteinuria and glomerular filtration rate were recorded at diagnosis and during the following 6-months to assess the clinical course, response and remission rates. We hope knowledge of the typical renal outcome will lead to more evidence-based treatment algorithms.

## Methods

### Study design

This descriptive retrospective cohort study is based on the German Henoch-Schönlein purpura nephritis register, supported by the German Society of Pediatric Nephrology (GPN). All patients were enrolled from seven pediatric nephrology departments (Munich, Cologne, Hamburg, Berlin, Erlangen, Muenster and Freiburg). Inclusion criteria were age 0–18 years, purpura before onset of nephritis and biopsy-proven IgAVN including mesangial IgA-deposition on kidney biopsy. Patients with comorbidities, chronic illness or prior kidney disease were excluded, resulting in a total cohort of 66 patients. Information about the initial onset of nephritis as well as all follow-up visits during the first 6 months (month #1 (M1) – month #6 (M6)) was collected on site by review of the patient files. The study complied with the Declaration of Helsinki and was approved by the ethics committee of the Albert Ludwig’s University of Freiburg, Germany.

### Definitions, clinical and laboratory parameters

Age, gender, five main clinical features (purpura, edema, arthritis, abdominal pain and hypertension) and hematuria (macroscopic or microscopic) were documented. In terms of laboratory results we noted the highest level of proteinuria [urinary protein/creatinine ratio in g/g or g/m^2^/day in 24 h-urine sampling] and serum creatinine [mg/dl] and the lowest level of serum albumin [mg/dl] and glomerular filtration rate [ml/min/1.73m^2^]. The estimated glomerular filtration rate (eGFR) was calculated with the modified Schwartz formula [16]. Nephrotic-range proteinuria was defined as > 2.0 g/g creatinine in spot urine sampling or > 1.0 g/m^2^/day in 24 h-urine sampling. Non-nephrotic range proteinuria was defined as urinary loss of protein between 0.2 – 2.0 g/g creatinine or 0.15 – 1.0 g/m^2^/day respectively. The cut-off for impaired eGFR was specified as < 90 ml/min/1.73m^2^. Serum albumin levels were considered normal between 35–55 mg/dl. Nephrotic syndrome was defined as nephrotic-range proteinuria with either decreased serum albumin < 25 mg/dl or edema. Nephritic syndrome was diagnosed when hematuria and a decrease of eGFR were present. Hypertension was assessed by the local attending physician. Complete remission was defined as eGFR > 90 ml/min/1.73m^2^ and no proteinuria (< 0.2 g/g creatinine or < 0.15 g/m^2^/day).

### Histology

Results of the kidney biopsy were obtained from the local pathologists’ reports. We evaluated proportion and type of crescents as well as chronic lesions. Cellular and fibrocellular crescents were classified as acute lesions. On the contrary, fibrous crescents, > 5% tubular atrophy, > 5% tubulointerstitial fibrosis and glomerulosclerosis were categorized as chronic lesions.

### Statistics

Variables were tested for normal distribution using the Shapiro–Wilk test. Normally distributed variables were compared using Student’s t-test, whereas the Wilcoxon signed-rank test was used when variables were not normally distributed. Results are given as median and interquartile range (IQR). The chi-square test was applied for dichotomous features. *P*-values < 0.05 were considered significant.

## Results

### Patient characteristics and parameters at onset of diagnosis

Sixty-six patients with biopsy-proven IgAVN were included in the study. Diagnosis was confirmed between March 1999 and March 2012. 33 (50%) were female and 33 (50%) male. Median age at diagnosis was 8.9 (IQR 6.1–11.4) years. Patient characteristics at onset of nephritis are given in Table 1.

**Table 1:** Patient characteristics of 66 patients with biopsy-proven IgA vasculitis nephritis (IgAVN) at onset of nephritis. The number of patients varies because of missing values

	median (interquartile range)	
Age at diagnosis of IgAVN (*n* = 66)	8.9 (6.1–11.4) years	
Days from IgAV to first nephritic symptoms (*n* = 66)	11.5 (1–31) days	
Days from first nephritic symptoms to biopsy (*n* = 66)	30 (9–57) days	
	n	%
**Clinical symptoms** (*n* = 66)		
Edema	22	30.3
Hypertension	15	22.7
Purpura	62	93.9
Arthritis	25	37.9
Abdominal pain	41	62.1
**Serum albumin**		
Decreased serum albumin (*n* = 51)	32	62.8
Median serum albumin (*n* = 51)	33 (30–39.2) mg/dl	
**Proteinuria**		
Non-nephrotic-range proteinuria (*n* = 61)	10	16.4
Nephrotic-range proteinuria (*n* = 61)	49	80.3
No proteinuria (*n* = 61)	2	3.3
Median proteinuria (*n* = 59)	3.7 (1.9 – 6.4) g/g creatinine	
**eGFR**		
Impaired renal function (*n* = 64)	32	50.0
Median eGFR (*n* = 63)	86.7 (75.3 – 118.0) ml/min/1.73m^2^	
**Hematuria**		
Microscopic hematuria (*n* = 65)	62	95.4
Macroscopic hematuria (*n* = 65)	20	30.8
Nephrotic syndrome (*n* = 64)	9	14.1
Nephritic syndrome (*n* = 64)	24	37.5
Nephrotic-nephritic syndrome (*n* = 64)	8	12.5

### Treatment

**Fig. 1:**
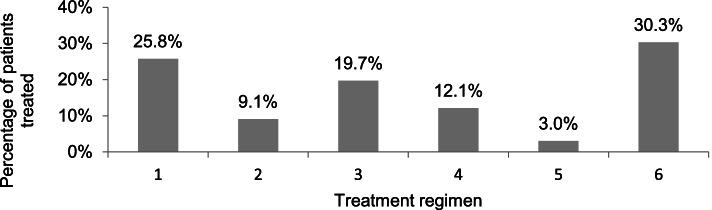
Proportional distribution of different immunosuppressive therapy regimens. 1) corticosteroid pulse therapy + maintenance therapy with oral corticosteroids. 2) oral corticosteroids only. 3) corticosteroid pulse therapy + maintenance therapy with oral corticosteroids + other immunosuppressants. 4) oral corticosteroids + other immunosuppressants. 5) other immunosuppressants only. 6) no immunosuppressants

The treatment regimens of our 66 patients varied notably (Fig. 1). Throughout the 6 months of the follow-up period, 69.7% (*n* = 46/66) of the patients were treated with immunosuppression. Among these, CS, MMF, CYC and CSA were used in various combinations. 90.9% (*n* = 60/66) received angiotensin converting enzyme (ACE-) inhibitors, 9.1% (*n* = 6/66) had angiotensin II receptor subtype 1 (AT1-) antagonists additionally. 17 patients with crescents or nephrotic syndrome were treated with CS pulse therapy (3 × 300 mg/m^2^ every 48 h), followed by a CS maintenance therapy (60 mg/m^2^ for 3 weeks, 40 mg/m^2^ for another 4 weeks) according to the recommendations of the GPN. 11 patients were not treated with immunosuppressive therapy despite the presence of crescents in their kidney biopsy.

### Histology

**Fig. 2:**
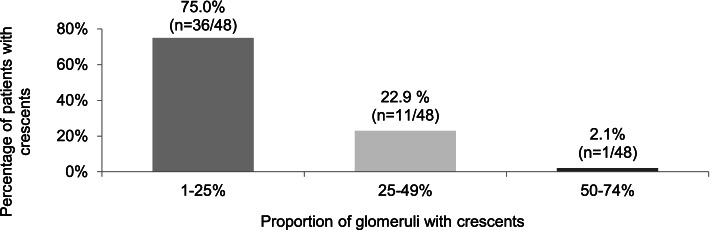
Proportional distribution of glomeruli with crescents (cellular, fibrous, fibrocellular)

According to the inclusion criteria, all patients (*n* = 66/66) showed mesangial IgA-depositions on kidney biopsy. A median of 19 (IQR 13–34) glomeruli per biopsy sample were analysed. 72.7% (*n* = 48/66) of the patients showed crescents in their biopsy, a median of 18.8% (IQR 11.3 – 24.1) glomeruli were affected by these findings. The proportion of crescents is illustrated in Fig. 2. None of the patients presented with crescents in more than 75% of the glomeruli of the kidney biopsy. Cellular crescents were described in 70.8% (*n* = 34/48) of these cases, fibrocellular crescents in 10.4% (5/48) and fibrous crescents in 12.5% (*n* = 6/48). However, in 20.8% (*n* = 10/48) no further specifications were made regarding the type of crescents. Chronic lesions were present in 48.5% (*n* = 32/66) of the patients comprising fibrous crescents, > 5% tubular atrophy, > 5% tubulointerstitial fibrosis or glomerulosclerosis.

### Six-months clinical course

**Fig. 3:**
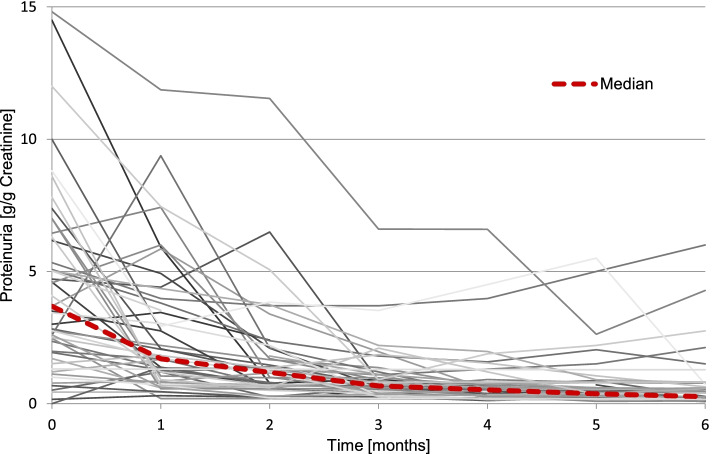
Six-month follow-up of proteinuria in 66 patients with biopsy-proven IgAVN. Median proteinuria dashed thick red line (-—-). Threshold heavy proteinuria thick yellow line (ꟷ·ꟷ·)

A median of 4.6 (range: 1—10) outpatient visits were recorded during the first six months of follow-up. Hospital admission was necessary in 20 patients (30.3%) during that time. During the first 3 and 6 months all five clinical symptoms (purpura, edema, hypertension, arthritis, abdominal pain) decreased significantly in comparison to the initial presentation (*p* < 0.01 in all 3- and 6-months values vs. onset). Table 2 illustrates the improvement of renal impairment and the decrease of proteinuria as well as the decreasing proportion of patients with nephrotic-range proteinuria and the increase of patients achieving remission. Median proteinuria decreased significantly within 3 and 6 months, as did the proportion of patients with nephrotic-range proteinuria. Within the group of patients with heavy proteinuria at onset of disease, proteinuria dropped in greater extent compared to patients with initial non-nephrotic range proteinuria (from 5 to 0.25 g/g Creatinine vs. 1 g/g to 0.4 g/g Creatinine after 6 months). Figure 3 visualizes the major decrease of median proteinuria within the first 2 months. After 4 months, two patients (*n* = 2/52) and after 6 months more than one quarter of the patients (*n* = 13/49) had achieved complete remission. When comparing groups with different therapeutic regimens, no significant impact on the course of the nephritis was found. Treated and untreated patients with similar histological and clinical presentation showed similar improvement of clinical symptoms and proteinuria (see additional file 1), but the patient number was too small for a meaningful data subanalysis.

**Table 2:** Changes of eGFR and proteinuria of the total cohort during the first 6 months of follow-up. *P* values are shown for the difference between the values at month 3 (M3) and month 6 (M6) versus the values at onset

	Onset of disease	M1	M2	M3	***p***	M4	M5	M6	***p***
Median eGFR [ml/min/1.73m^2^]	86.7	101.1	101.4	98.0	*0.214*	103.6	105.0	101.6	*0.190*
eGFR < 90 ml/min/1.73m^2^ [%]	50.0	35.1	39.7	34.9	*0.449*	44.4	34.6	35.5	*0.106*
Median proteinuria [g/g creatinine]	3.7	1.7	1.2	0.7	** < *0,001***	0.5	0.4	0.3	** < *0,001***
Nephrotic-range proteinuria [%]	80.3	40.4	25.9	11.5	** < *0,001***	6.3	9.8	7.0	** < *0,001***
Non-nephrotic range proteinuria [%]	16.4	56.3	70.0	89.6	** < *0,001***	93.5	89.8	92.5	** < *0,001***
No proteinuria [%]	0	0	0	0		8.3	18.0	30.9	** < *0,001***
Remission [%]	0	0	0	0		3.9	13.5	27.1	** < 0,001**

## Discussion

In our cohort all clinical symptoms (purpura, edema, hypertension, arthritis, abdominal pain) as well as the proportion of patients with macroscopic hematuria and nephrotic syndrome decreased significantly within 3 months. Proteinuria was reduced significantly as well within this time frame. Kidney function increased, albeit without achieving the level of significance within 6 months. After a period of 6 months 26.5% of the children were in complete remission. Prior studies rarely described the short-term course of IgAVN in detail and most referred to specific treatment regimens, so that it is difficult to base clinical treatment decisions on the published literature. Treatment in the patient cohort was highly variable which complicates comparison between different regimens. Between the small subgroups no significant impact of the treatment decisions could be shown. The majority of the reports published to date focused on corticosteroids intravenously or orally, often combined with other immunosuppressive drugs. In adults, a beneficial effect of corticosteroids has been postulated in patients with IgAN [17, 18], but they did not improve the outcome in a large randomized trial [19, 20]. In children with IgAVN there are no large randomized studies available. As illustrated in detail in Table 3 and discussed below, several previous studies described the short-term follow-up at specific time points using specified therapeutic regimens, whereas our study depicts the individual time course of proteinuria and kidney function after the diagnosis of IgAVN regardless of the therapy.

**Table 3:** Published literature referring to short-term clinical course in children with IgAVN

Study	Study design	Number of patients	Therapy	Median age and range (years)	Severity of disease at onset	Short term follow-up
**Corticosteroids**						
Kawasaki et al. [13]	Retrospective	*n* = 56 *n* = 56 biopsied	*n* = 56 CS pulse therapy + urokinase pulse therapy, dipyridamol and warfarin	8.6 ± 2.9* *not specified whether median/mean	*n* = 31 proteinuria *n* = 25 nephrotic syndrome *n* = 10 renal insufficiency	6 months decrease of proteinuria, *n* = 0 renal insufficiency
Deng et al. [21]	Retrospective	*n* = 186 number of renal biopsies unclear	*n* = 21 no immunosuppressant *n* = 105 hydrocortisone sodium succinate *n* = 36 CS pulse therapy *n* = 24 CS pulse therapy, tripterygium glycoside *n* = 186 ACE inhibitors	9.56 – 10.25 depending on group* *not specified whether median/mean	*n* = 183 proteinuria	4 weeks *n* = 113 decrease of proteinuria
Niaudet et al. [14]	Prospective	*n* = 38 *n* = 38 biopsied	*n* = 31 CS pulse therapy *n* = 7 CS pulse therapy + CYC	7.7 (3.0–14.2)* *mean age	*n* = 36 nephrotic syndrome *n* = 3 renal insufficiency	5 months decrease of proteinuria, *n* = 12 resolution of proteinuria, *n* = 0 renal insufficiency
**Cyclophosphamide**						
Wakaki et al. [22]	Retrospective	*n* = 42 *n* = 33 biopsied	*n* = 17 no immunosuppressants *n* = 25 CS alone or CS + CYC + AZA and/or CS pulse therapy	7.4 (2.8–14.2)	*n* = 42 nephrotic-range proteinuria *n* = 16 renal insufficiency	< 3 months *n* = 19 resolution of nephrotic-range proteinuria, *n* = 17 complete clinical remission
Iijima et al. [23]	Retrospective	*n* = 14 *n* = 14 biopsied	*n* = 14 CYC + CS, heparin, warfarin, dipyridamol	6.7 (5.0–17.5)	*n* = 7 heavy proteinuria *n* = 1 nephrotic syndrome *n* = 2 nephritic syndrome *n* = 3 nephrotic-nephritic syndrome	4–5 months *n* = 1 heavy proteinuria, *n* = 0 renal insufficiency
Flynn et al. [24]	Retrospective	*n* = 12 *n* = 12 biopsied	*n* = 4 oral CS + CYC *n* = 8 CS pulse therapy + CYC	8.6 (5.9–15.3)	*n* = 12 nephrotic-range proteinuria	3 months *n* = 12 decrease of protein-to-creatinine ratio
Kawasaki et al. [25]	Retrospective	*n* = 37 *n* = 37 biopsied	*n* = 20 CS pulse therapy + urokinase pulse therapy *n* = 17 CYC additionally	7.9 – 8 depending on group	*n* = 26 nephrotic-range proteinuria *n* = 16 renal insufficiency	6 months Decrease of proteinuria, *n* = 0 renal insufficiency *n* = 21 fully recovered
**Cylosporine A**						
Park et al. [26]	Retrospective	*n* = 29 *n* = 6 biopsied	*n* = 29 CS + CSA *n* = 29 ACE inhibitors	8.6 (2.0–15.5)	*n* = 6 nephrotic syndrome *n* = 23 nephrotic-range proteinuria *n* = 1 renal insufficiency	1.8 months Resolution of proteinuria at a mean of 1.8 months (1 week to 3.5 months)
Jauhola et al. [27]	Randomized controlled	*n* = 24 *n* = 24 biopsied	*n* = 11 CSA *n* = 13 CS pulse therapy *n* = 24 ACE inhibitors	9.4 (4–16)	*n* = 24 nephrotic-range proteinuria or crescents	3 months *n* = 18 remission of nephrotic-range proteinuria
Ronkainen et al. [28]	Prospective	*n* = 7 *n* = 7 biopsied	*n* = 7 CSA *n* = 7 ACE inhibitors	10.6 (7.2–15.2)	*n* = 7 nephrotic-range proteinuria	1.4 months Decrease of proteinuria at a mean of 1.4 months (1 week to 4 months)
**MMF**						
Du et al. [29]	Retrospective	*n* = 12 *n* = 12 biopsied	*n* = 12 oral CS, CS pulse therapy, MMF, heparin, dipyridamol *n* = 12 ACE inhibitors	8.33 (6–12)	*n* = 12 nephrotic-range proteinuria	3 months *n* = 12 decrease of proteinuria to a minimum of 50% of pretreatment level
Ren et al. [30]	Retrospective	*n* = 53 *n* = 53 biopsied	*n* = 26 oral CS *n* = 27 MMF	27 (14–62)	*n* = 53 heavy proteinuria *n* = 4 renal insufficiency	6 months *n* = 42 remission
**Azathioprin**						
Ninchoji et al. [31]	Retrospective	*n* = 50 *n* = 50 biopsied	*n* = 31 ACE inhibitors and/or AT1-antagonist *n* = 19 oral CS + AZA/mizoribin, warfarin	8.73 ± 0.54* *not specified whether median/mean	*n* = 31 moderately severe IgAVN (ISKDC I-III) *n* = 19 severe IgAVN (ISKDC IV-V)	4.3 months 50% resolution of proteinuria in moderately severe IgAVN 5.2 months 50% resolution of proteinuria in severe IgAVN
Altugan et al. [32]	Prospective	*n* = 18 *n* = 18 biopsied	*n* = 18 Oral CS ± CYC ± AZA *n* = 18 ACE inhibitors	11.2 ± 4.0* *mean age	*n* = 1 nephrotic syndrome *n* = 4 nephritic syndrome *n* = 13 nephritic syndrome + heavy proteinuria *n* = 7 renal insufficiency	4–6 months *n* = 18 resolution of proteinuria

In contrast to the recently established European SHARE guideline [33] the recommendation of the German Society of Pediatric nephrology advocates an early biopsy [34]. Therefore, the patients in this cohort were biopsied relatively early and even in these early biopsies chronic changes were found in a significant percentage of patients. It appears that irreversible damage in IgAVN occurs either rapidly or the disease had been preexisting, so that early biopsies might be justified to estimate the individual patient prognosis.

The most profound improvement of renal impairment in our cohort was seen within one to two months of follow up which is consistent with a cohort published by Kawasaki et al., treated with a cyclophosphamide containing regimen [25]. Deng et al. evaluated treatment effects after an interval of four weeks [21]. Different therapeutic regimens were used in this study, ranging from non-steroid strategies to hydrocortisone sodium succinate, methylprednisolone pulse therapy and methylprednisolone in combination with tripterygium glycoside. While 60.8% of their patients presented decreasing proteinuria or no hematuria within this interval, 39.2% were stratified as non-responders and therefore received intensified treatment [21]. Our cohort clearly shows that patients continue to improve after the first four weeks. In another study by Ronkainen et al., seven patients with nephrotic-range proteinuria received CSA and were observed monthly for the first six months. All patients showed a certain response within a mean time of 1.4 months [28].

At 3 months, Niaudet et al. analysed children with biopsy-proven IgAVN treated with corticosteroids in an uncontrolled study. Similar to our cohort, their 38 patients showed a significant decrease in proteinuria within that timeframe [14]. Three cohorts receiving cyclophosphamide demonstrated similar results [22–24]. Cyclophosphamide in combination with corticosteroids was the therapy of choice in two of these cohorts [23, 24] whereas Wakaki et al. evaluated a heterogenous group of patients stratified by the International Study of Kidney Disease in Children (ISKDC) classification. Grade I-III were not treated with immunosuppressants, grade IV-V treated with corticosteroids with or without CYC, AZA and/or methylprednisolone pulse therapy [22]. Resembling our data, a significant reduction of proteinuria has been noticed [24]. Evaluation of the duration of proteinuria showed a reduction of proteinuria to non-nephrotic levels in 45.2% within 3 months [22]. In our cohort, the proportion of patients with nephrotic-range proteinuria decreased significantly from 80.3% to 11.5% within 3 months. Two groups of patients treated with CSA have been analysed after a period of 3 months [26, 27]. Jauhola et al. analysed 24 patients with at least ISKDC grade III and randomized them into a group treated with CSA and a group treated with methylprednisolone. Remission of nephrotic-range proteinuria has been achieved in all of CSA patients, whereas the remission rate of the control group was inferior and slower [27]. The second cohort received CSA when nephrotic-range proteinuria evolved during oral corticosteroid treatment. A response (defined as trace to negative proteinuria) was noted within 3.5 months in all patients. A small cohort treated with MMF after being found steroid-resistant demonstrated a mean response interval of 2.5 months with decreasing proteinuria to a minimum of 50% compared to pretreatment parameters [29].

At 4- and 5-months follow-up, Ninchoji et al. published 50 patients with moderate or severe IgAVN without immunosuppressive treatment and with prednisone and azathioprine or mizoribine respectively. Resolution of proteinuria was achieved in 50% of the patients by 4.3 months in those with moderate IgAVN and by 5.3 months in those with severe IgAVN [31]. Remission of proteinuria was obtained in a group of 18 patients treated according to their ISKDC grade with oral prednisolone with and without CYC with and without AZA by 4–6 months; the authors therefore discuss an escalating treatment algorithm according to severity [32].

A half-year follow-up was published in two heterogeneous patient cohorts [25, 30]. Kawasaki et al. evaluated mean UPE of 37 patients treated with either urokinase pulse therapy or urokinase pulse therapy plus cyclophosphamide, whereas Ren et al. looked at 53 patients who received MMF or prednisone. Proteinuria was decreased significantly in all groups at 6 months follow-up [25].

The short-term renal course of IgAVN over the first 6 months after kidney biopsy appears benign with regression of proteinuria and improvement of renal impairment in the overwhelming number of patients, but in our cohort complete remission was not observed before 3 months and is reached after 6 months in a minority of the patients. The data we present shows the typical initial course of IgAVN with current treatment after kidney biopsy. Decisions for or against aggressive immunosuppressive therapy should be based on this typical disease course and lacking improvement after 4 weeks not necessarily requires additional, more aggressive treatment.

## Conclusion

In conclusion, we see improvement of kidney function and decreasing proteinuria in all patients in within the first months, but complete remission requires more time in most patients. Persistent heavy proteinuria in the first two months rarely developed into long-term proteinuria. Therefore, decisions for more intense treatment should take into account the course of UPE over time. Due to the retrospective design of the study, the effect of different immunosuppressive therapy regimens cannot be compared, but treated and untreated patients showed a similar time course of proteinuria.

For clarifying the effect of immunosuppressive therapy in IgAVN, prospective, randomized controlled multicenter trials are necessary.

## Supplementary Information

**Additional file 1.** Comparative subgroup analysis. Of 66 patients, we identified 28 children with crescentic glomeruli and/or nephrotic syndrome at onset of IgAVN. Referring to the German Society of Pediatric Nephology, a corticosteroid pulse therapy only would have been recommended. Of these 28 children, 17 received corticosteroid pulse therapy per protocol, 11 did not receive any immunosuppressive therapy. We therefore performed a comparative subgroup analysis which showed similar improvements of clinical symptoms and proteinuria.

## Data Availability

The datasets used and/or analysed during the current study are available from the corresponding author on reasonable request.

## Abbreviations

IgAV: IgA vasculitis
IgAVN: IgA vasculitis nephritis
UPE: Urinary protein excretion
eGFR: Estimated glomerular filtration rate
CS: Corticosteroids
MMF: Mycophenolate mofetil
CSA: Cyclosporine A
CYC: Cyclophosphamide
AZA: Azathioprine
ACE: Angiotensin converting enzyme
AT1: Angiotensin II receptor subtype 1
IQR: Interquartile range
ISKDC: International Study of Kidney Disease in Children
