# Supplementary material for: Early clinical course of biopsy-proven IgA vasculitis nephritis
Source: BMC Pediatr. 2022 Oct 4;22:570. doi: 10.1186/s12887-022-03611-9 (PMC9531371; doi:10.1186/s12887-022-03611-9)
Supplement: Supplementary file 1 — Additional file 1. Comparative subgroup analysis. Of 66 patients, we identified 28 children with crescentic glomeruli and/or nephrotic syndrome at onset of IgAVN. Referring to the German Society of Pediatric Nephology, a corticosteroid pulse therapy only would have been recommended. Of these 28 children, 17 received corticosteroid pulse therapy per protocol, 11 did not receive any immunosuppressive therapy. We therefore performed a comparative subgroup analysis which showed similar improvements of clinical symptoms and proteinuria. [file 12887_2022_3611_MOESM1_ESM.docx]

Additional file 1

**Comparative subgroup analysis**

Of 66 patients, we identified 28 children with crescentic glomeruli and/or nephrotic syndrome at onset of IgAVN. Referring to the German Society of Pediatric Nephrology, a corticosteroid pulse therapy only would have been recommended. Of these 28 children, 17 received corticosteroid pulse therapy per protocol, 11 did not receive any immunosuppressive therapy. We therefore performed a comparative subgroup analysis which showed similar improvements of clinical symptoms and proteinuria.

Group I: All patients with IgAVN and crescentic glomeruli or nephrotic syndrome treated with CS pulse therapy

Group II: All patients with IgAVN and crescentic glomeruli or nephrotic syndrome without immunosuppressive therapy

|  | I | II | *p* |
| --- | --- | --- | --- |
| Age at diagnosis of IgAVN (median) in years | 8.1  (3.9 – 16.9) | 7.6  (2.7 – 17.3) | *0.94* |
| Male | 11 | 5 | *0.32* |
| Female | 6 | 6 | *0.32* |
| Days from IgAV to first nephritic symptoms | 17 | 31 | *0.96* |
| Days from first nephritic symptoms to biopsy | 24 | 43 | *0.50* |

Table 1: Patient characteristics of two comparative groups (Group I: Patients with IgAVN and crescentic glomeruli or nephrotic syndrome treated with CS pulse therapy; Group II Patients with IgAVN and crescentic glomeruli or nephrotic syndrome without immunosuppressive therapy)

|  | Crescentic glomeruli [%] | 1-24% crescentic glomeruli [%] | 25-49% crescentic glomeruli [%] | ≥50% crescentic glomeruli  [%] | Chronic lesions  [%] |
| --- | --- | --- | --- | --- | --- |
| I (n=17) | 15,4 | 86,7 | 13,3 | 0 | 52,9 |
| II (n=11) | 18,8 | 72,7 | 27,3 | 0 | 36,4 |
| *p* | *0.28* | *0.37* | *0.37* |  | *0.39* |

Table 2: Histological findings of group I and II at onset of IgAVN

|  | Proteinuria  [g/g Creatinine]  (median) | Proteinuria  >2 g/g Creatinine  [%] | eGFR [ml/min/1.73m²]  (median) | eGFR  <90 ml/min/1.73m²  [%] | Nephrotic syndrome  [%] |
| --- | --- | --- | --- | --- | --- |
| I (n=17) | 4.7 | 82,4 | 81,0 | 70,6 | 29,4 |
| II (n=11) | 4.5 | 81,8 | 93,5 | 36,4 | 30,0 |
| *p* | *0.26* | *0.97* | *0.24* | *0.07* | *0.97* |

Table 3: Patient characteristics of group I and II at onset of IgAVN

|  | Proteinuria  [g/g Creatinine]  (median) | Proteinuria  >2 g/g Creatinine  [%] | eGFR [ml/min/1.73m²]  (median) | eGFR  <90 ml/min/1.73m²  [%] | Nephrotic syndrome  [%] |
| --- | --- | --- | --- | --- | --- |
| I (n=17) | 0,2 | 6,3 | 103,1 | 25,0 | 0 |
| II (n=11) | 0,4 | 0 | 102,7 | 30,0 | 0 |
| *p* | *0.67* | *0.40* | *0.58* | *0.78* |  |

Table 4: Patient characteristics of group I and II at 6 months follow-up

|  | Decrease of proteinuria  [g/g Creatinine]  (median) | Increase of eGFR [ml/min/1.73m²]  (median) |
| --- | --- | --- |
| I (n=17) | 4,5 | 28,0 |
| II (n=11) | 3,6 | 10,6 |
| *p* | *0.24* | *0.12* |

Table 5: Decrease of proteinuria and increase of eGFR in group I and II from onset of IgAVN until 6 months follow-up
